# Toll-Like Receptors: General Molecular and Structural Biology

**DOI:** 10.1155/2021/9914854

**Published:** 2021-05-29

**Authors:** Payam Behzadi, Herney Andrés García-Perdomo, Tomasz M. Karpiński

**Affiliations:** ^1^Department of Microbiology, College of Basic Sciences, Shahr-e-Qods Branch, Islamic Azad University, Tehran, Iran; ^2^Division of Urology. Department of Surgery, School of Medicine, UROGIV Research Group, Universidad del Valle, Cali, Colombia; ^3^Chair and Department of Medical Microbiology, Poznań University of Medical Sciences, Wieniawskiego 3, 61-712 Poznań, Poland

## Abstract

**Background/Aim:**

Toll-like receptors (TLRs) are pivotal biomolecules in the immune system. Today, we are all aware of the importance of TLRs in bridging innate and adaptive immune system to each other. The TLRs are activated through binding to damage/danger-associated molecular patterns (DAMPs), microbial/microbe-associated molecular patterns (MAMPs), pathogen-associated molecular patterns (PAMPs), and xenobiotic-associated molecular patterns (XAMPs). The immunogenetic molecules of TLRs have their own functions, structures, coreceptors, and ligands which make them unique. These properties of TLRs give us an opportunity to find out how we can employ this knowledge for ligand-drug discovery strategies to control TLRs functions and contribution, signaling pathways, and indirect activities. Hence, the authors of this paper have a deep observation on the molecular and structural biology of human TLRs (hTLRs).

**Methods and Materials:**

To prepare this paper and fulfill our goals, different search engines (e.g., GOOGLE SCHOLAR), Databases (e.g., MEDLINE), and websites (e.g., SCOPUS) were recruited to search and find effective papers and investigations. To reach this purpose, we tried with papers published in the English language with no limitation in time. The *iCite* bibliometrics was exploited to check the quality of the collected publications.

**Results:**

Each TLR molecule has its own molecular and structural biology, coreceptor(s), and abilities which make them unique or a complementary portion of the others. These immunogenetic molecules have remarkable roles and are much more important in different sections of immune and nonimmune systems rather than that we understand to date.

**Conclusion:**

TLRs are suitable targets for ligand-drug discovery strategies to establish new therapeutics in the fields of infectious and autoimmune diseases, cancers, and other inflammatory diseases and disorders.

## 1. Introduction

The mathematical and computational immunology shows the dynamics, kinetics, molecular and structural models, and characteristics of immune molecules, cells, signaling pathways and responses, and their synergistic mechanisms and cross-talks. The computational software tools enable us to have a strong understanding of immune responses and the pivotal role of important biomolecules like Toll-like receptors (TLRs) [1, 2]. The use of computational and mathematical models gives us a novel illustration of different parts of immunology.

TLRs as functional and effective biomolecules are pioneers on the front line of the immune defense system in different types of hosts from insects to mammals like humans [3–8]. As the immune system gate-keepers, TLRs, whether in humans or other mammals, are transmembrane proteins with conserved structures and evolutionary changes with a dual function within innate and adaptive immune systems [8]. In recent years, the soluble forms of TLRs (sTLRs) including sTLR2 and sTLR4 have also been characterized [9–11]. The sTLRs are detectable in different body fluids, e.g., blood, saliva, plasma, cerebrospinal fluid (CSF), amniotic fluid, breast milk, and pleural fluid [10–20].

The extracellular sTLRs are known as the main modulators which contribute to TLRs signaling pathways with negative regulating mechanisms (via antagonizing the proinflammatory activity of cell surface TLRs). In other words, sTLRs act as baits to prevent the induction of TLR-ligand signaling pathways [12, 16, 21, 22]. According to previous studies, the sTLR2 and sTLR4 molecules reduce the inflammation process through the inactivation of proinflammatory responses associated with TLR molecules. It may result in some interferences between sTLRs regulatory mechanisms and endogenous ligands of TLRs [11]. The reported reports indicate that the sTLRs comprising sTLR2 and sTLR4 can be applied as diagnostic biomarkers. However, more investigations are needed to be done in this regard [11].

The biomolecules of TLRs belong to the pattern recognition receptors (PRRs) [23–27]. The TLRs mediate immune responses against four types of different molecular patterns comprising damage/danger-associated molecular patterns (DAMPs), microbial/microbe-associated molecular patterns (MAMPs), pathogen-associated molecular patterns (PAMPs), and xenobiotic-associated molecular patterns (XAMPs) [7, 8, 24, 28]. The majority of PRRs are categorized into five families [25]. PRRs are determined as the exterior, membranous, and cytosolic (interior) structures of the cells, and their categorization is based on their activity, position, specific ligands, and evolutionary associations. According to protein domain homology, PRRs are categorized into five families [24, 25] including TLRs, C-type lectin receptors (CLRs), AIM2-like receptors (ALRs), Retinoic acid-inducible gene (RIG)-I-like receptors (RLRs), and nucleotide-binding domain, leucine-rich repeat (LRR)-containing (NOD-like) receptors (NLRs). The first two families are transmembranes proteins and belong to membrane-bound receptors, while the second three families are cytoplasmic proteins and pertain to unbound intracellular receptors [7, 23, 25–27].

Indeed, TLRs and CLRs are responsible for determining their specific ligands upon the cell surfaces and within endosomes. Simultaneously, the molecules of ALR, NLR, and RLR as the cytosolic proteins are responsible for detecting intracellular pathogens [25]. In addition to the aforementioned five families of PRRs, recently (in 2013), the sixth family of PRRs has been identified. The sixth one is named as cyclic guanosine monophosphate- (GMP-) adenosine monophosphate (AMP) synthase (cGAS) and is detectable in both cellular sections of cytosol and the nucleus [29]. The cGAS as a member of the nucleotidyltransferase family is capable to identify the cytosolic DNAs and the extracellular nucleosomes. It also triggers the expression of type I interferons (IFNs) [29–31]. Furthermore, the stimulator of IFN genes (STING) is identified as an endoplasmic reticulum (ER) adaptor which is associated with the cGAS signaling pathway [29, 32, 33]. Indeed, the cGAS-STING signaling pathway act as cytosolic PRRs to identify intracellular double-stranded DNA (dsDNA) molecules and inducing the expression of type I IFN in the presence of dsDNA viruses, host-originated self dsDNAs, and the retroviruses of human immunodeficiency virus-1 (HIV-1) and HIV-2. The optimal length of dsDNA molecules which can be detected by cGAS is ≥36 bp [29, 34–36].

The PRRs, which are known as innate immune receptors, are detectable upon the cell surfaces within the endosomes, cytoplasm, and serum Botos and Segal [37]. In accordance with our knowledge, the major and minor portions of innate immune responses triggered by PRRs are transcriptional and nontranscriptional, respectively. The transcriptional mediators are comprised of IFNs and proinflammatory cytokines, while the nontranscriptional mediators contribute in triggering processes of autophagy, cell death, cytokine processing, and phagocytosis [25, 38–40]. The TLR glycoproteins as a family member belonging to PRRs are involved in the expression of transcriptional mediators which may lead to produce IFNs and proinflammatory cytokines [25, 28, 38–40]. The biomolecular sensors of TLRs as members of the type I transmembrane receptors are composed of three vital segments comprising an extracellular ligand-binding structure known as ectodomain or N-terminal ligand recognition domain with a folded structure of solenoid involving tandem short motifs of Leucine-rich repeats (LRRs), a single transmembrane fragment with a single helix configuration and an intracellular C-terminal domain (CTD) of Toll/IL-1R (TIR) homology as a cytosolic signaling segment [37, 41, 42].

**Figure 1:**
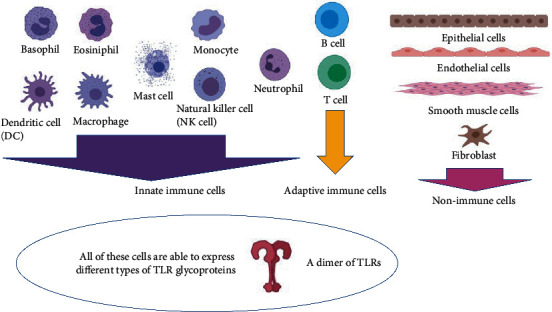
Different types of immune and nonimmune cells are able to express different types of TLR glycoproteins (this figure is prepared via the software tool serving by http://Biorender.com/).

The ligand detection occurs in the outer segment of TLRs. Simultaneously, the induction of effective immune responses is achieved by the interior segment of TLRs (TIR domains) through the activation of the complex signal network via employing adaptors, e.g., myeloid differentiation primary response protein 88 (MyD88), to begin the cascades of the innate immune system [8, 43]. The Germline encoded molecules of TLRs are expressed by different immune cells (usually by innate immune cells and lesser by adaptive immune cells), nonprofessional cells, and nonimmune cells. Depending on the type of TLR molecules, they can be expressed by basophils, dendritic cells (DCs), eosinophils, macrophages (M*Φ*s), mast cells, monocytes, natural killer (NK) cells, neutrophils, lymphocytes of B- and T cells, epithelial cells, endothelial cells, fibroblasts, and smooth muscle cells (Figure 1) [6, 7, 23, 27, 28, 42, 44–49].

Up to date, 10 (1-10) and 12 (1-9, 11-13) functional TLRs are identified in humans and mice, respectively. The *TLR10* gene in mouse genome is the result of retrovirus insertion, and therefore, the *TLR10* is known as an inactive pseudogene in mouse [6, 7, 23, 26, 28]. In contrast, *TLR11*, *TLR12*, and *TLR13* genes have been deleted from human's genomic pool. Based on TLR molecules amino acid sequences, the family of TLRs in human (hTLRs) consists of five members such as TLR1, TLR3, TLR4, TLR5, and TLR7 (Table 1) and in mice is comprised of seven members including TLR1, TLR3, TLR4, TLR5, TLR7, TLR11, and TLR13 [7, 23, 26, 28, 50, 51]. These biosensors have their specific molecular structures, characteristics, and abilities and are involved in infectious diseases, autoimmune diseases, and even cancers. Hence, in this review, we represent our understanding of the molecular and structural biology of TLRs.

## 2. Toll-Like Receptors (TLRS): History and Evolution

TLRs are known as the compartments of the TIR superfamily. Although the TLR glycoproteins are known as the ancient family of the PRRs with conserved structures from Porifera (invertebrate animals, e.g., sponges) to mammals, in 1985, the first Toll (Toll-1) was discovered in *Drosophila melanogaster* [3, 52, 53]. In accordance with previous reports, the complements system (CS) as an early compartment of the innate immune system (with a billion-year evolutionary background) is even older than TLRs [16, 54]. The evidences are obtained from single-cell microorganisms, e.g., choanoflagellates. These microorganisms are armed with CS but not TLRs. In brief, the CS is an old PRR with limited protectivity against pathogens; hence, the TLRs got evolved over the time as effective multifunctional PRRs against a wide range of pathogenic microorganisms [16, 55, 56]. TLRs' critical roles in different organisms are to detect different antigens (self and nonself) and cytokines' mediation to bridge innate and adaptive immune systems together [28, 53].

In accordance with the presence and the number of cysteine clusters within the extracellular domain of TLRs—LRR motif—the primary or prototypical TLRs are categorized into two groups of Protostome type (P-type) or Multiple Cysteine Cluster TLR (mccTLR) and Vertebrate type (V-type), prototypical type or Single Cysteine Cluster TLR (sccTLR), respectively [3, 50, 57, 58]. Those TLRs bearing a single cluster of cysteine or CF motif on their CTDs (LRRCT) are determined as V-type TLRs. In contrast, the P-type TLRs bear two or more (multiple) cysteine clusters or CF motifs on their LRRCT and sometimes a cysteine cluster on their N-terminal (LRRNT) domain (NTD) (NF motif). P-type or mccTLR seems to be rooted from the most ancient origins (only seen in invertebrates, including insects and nematodes) [3, 4, 57–60].

Simultaneously, the sccTLRs or V-type TLRs have been identified in vertebrates, e.g., mammals and some invertebrates [3, 50, 53, 58, 61]. *In toto*, about 10 TLR molecules and in particular, sccTLRs—classified as prototypical TLRs (V-type)—are identified among vertebrates [50, 58]. It is suggested that in contrast to V-type TLRs, the P-type TLRs bind to their ligands (MAMPs) indirectly [53], as it happens in *Drosophila* Toll. *Drosophila* Toll is not able to identify the microbial agents. The endogenous ligand (DAMP) of *Drosophila*, known as Späzle, mediates this recognition [3, 58]. Based on phylogenetic studies associated with TIR domain in TLRs, the sccTLRs (including V-type with classical sccTLR LRR structure, sP-type as shorter V-type structure, and Ls-type with no LRRNT domain and with noncanonical and degenerated LRR motifs) and mccTLRs (involving P-type with Drosophila Toll structure, sPP-type with the same structure of P-type LRR motif but in shorter scale, and Twin-TIR resembling P-type with two tandem TIR domains) are divided into three subtypes, respectively [57]. Up to date, 28 TLR molecules (TLR1-16, TLR18-28) are identified in vertebrates and 222 TLRs in invertebrates (e.g., the sea urchin of *Strongylocentrotus* belonging to Echinodermata). The lowest number of TLR molecules in invertebrates is reported as 1 (e.g., in the nematode of *Caenorhabditis elegans*). Mammals encompass maximally 13 TLR molecules, including humans (TLR1-10) and mice (TLR1-13) [23, 26, 28, 53, 62–64].

The highest number of TLRs with 21 TLR molecules comprising TLR1-5, 5S, TLR7-9, TLR13-14, TLR18-23, and TLR25-28 is recognized in the vertebrate fish of teleost. Noticeably, the TLRs 1-3, 5, and 7-9 in the teleost have the same characteristics in their activities and structures as they have in mammals [53, 58, 65–68]. Other TLRs in teleost have not close similarities with their family in mammals [53]. The outcome of the phylogenetic investigations indicates the presence of a certain ancestral *TLR* gene cluster, which independently has been evolved by versatile gene duplication, gene conversion, and coevolution over the millennia [43, 50, 58]. Moreover, TLR3 is the most ancient TLR family member and belongs to viral TLRs. Interestingly, the human viral TLRs (including TLR3, TLR7, TLR8, and TLR9 (known also as intracellular TLRs which bind to viral RNAs)) rather than nonviral TLRs (TLR1, TLR2, TLR4, TLR5, and TLR6 (known also as cell membrane TLRs which bind to PAMPs/MAMPs)) are much more purified on the evolutionary pathway (Table 1) [23, 26, 28, 50, 69–73].

The studies show that the origination of *TLR* genes goes back to >700 million years ago, detected in the ancestor of animals' phyla [58]. The ancient evidence reveals the absence of LRR motifs in the exterior section of TLR molecules. The first TLR molecules were only possessed the transmembrane domain and the cytosolic domain of TIR. Later, the independent extracellular structure of LRR motifs was added to the original molecules of TLRs [3, 58, 74]. Hence, the present form of TLR molecule is three-sectional with recognition ability of conserved patterns and downstream signaling activities [43, 58].

## 3. Leucine-Rich Repeats (LRRs)

LRRs are identified in more than 430,000 proteins in different organisms, from viruses to eukaryotes. The LRR proteins contribute in immune responses (both in plants and mammals innate immune responses), autophagy, type III secretion system (T3SS) belonging to bacterial pathogens, apoptosis, neural development, processes associated with ubiquitin, and nuclear mRNA transport [75–77]. The LRRs are proteins involving 20-40 amino acid residues, which usually are repeated in tandem and are rich in leucine (known as a hydrophobic amino acid). These repeated sequences are categorized into two types of the variable segment (VS) or highly conserved segment (HCS) [24, 75, 78–80]. The HCS portion usually involves 11 to 12 residues stretch including LxxLxLxxNxL or LxxLxLxxNxxL (in which L depicts Isoleucine, Leucine, Phenylalanine, or Valine; N depicts Cysteine, Asparagine, Serine, or Threonine; and x depicts any amino acid), and the novel HCSs include VxGxLxLxxNxL and VxGxLxLxxNxxl (in which G depicts Glycine; L depicts Cysteine, Leucine or Phenylalanine; N depicts Cysteine or Asparagine; V depicts Cysteine, Isoleucine, Leucine or Valine; and x depicts any amino acid) [24, 75, 79, 81].

**Figure 2:**
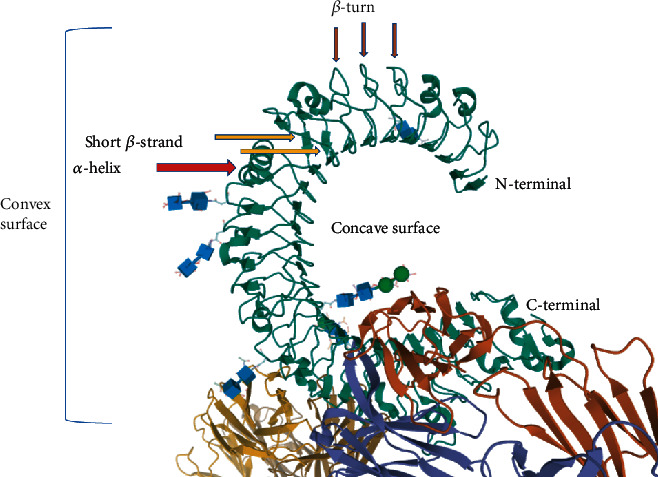
The horseshoe-like structure of LRR belonging to TLR3 ectodomain. The solenoid configuration of the LRR domain in TLR3 (3ULU PDB file) [85].

The canonical LRRs based on their VS portion characteristics including lengths and consensus sequences are categorized into nine classes of Bacterial (S), Cysteine-Containing (CC), CD42b-like (CD42b), Leptospira-like, Plant-Specific (PS), RI-like (RI), SDS22-like (SDS22), Typical (T), *Treponema pallidum* (Tp), and IRREKO [24, 75]. Today, by the help of bioinformatic software tools such as Pfam, InterPro, and LRRsearch SMART, we are able to predict the related LRR motifs [24]. The short *β*-strands build solenoids (superhelix structure) in the HCS portion of LRRs. The solenoid configuration is the result of the particular arrangement of short *β*-strands. They stack parallel with N-H→O=C or H-binding pattern at positions of three to five in LxxLxLxxNxL or LxxLxLxxNxxL sequences of HCS. The superhelical structure of solenoids creates different spatial structures, including horseshoe configuration (Figure 2), super helices with left or right-handed structures, and prism-like structures. The VS portion may include versatile secondary structures involving *α*-helix, 3(10)-helix, polyproline II helix (PPII), an extended structure, or *β*-turns in tandem, which are present in all classes of LRRs [75, 79, 82–84].

A solenoid of the LRR domain consists of four segments including convex and concave surfaces and ascending and descending loops [79, 83]. As Figure 2 shows, the horseshoe's inner side—which involves the HSC portion of the LRR domain—creates the concave surface where the short *β*-strands build the *β*-sheet configuration. In contrast, the outside of the concave surface forms the convex surface. The convex surface is consisted of VS portion of the LRR domain where several secondary structures, e.g., *α*-helices and unstructured loops, are involved [42, 79, 83].

The biophysical parameters of LRR radii and the *β*-sheet rotation and slope angles and the related twists govern the spatial configuration of *β*-sheets in LRR domains. Normally, the *β*-sheet angles match the radii of LRR domains Kang [83]. The bioinformatic investigations confirm the three-folded structure of the LRR domains in TLRs of 1, 2, 4, 6, and 10. In other words, TLR1 family members consist of three subdomains, including N- and C-termini and a central portion. This feature occurs in consequence of the presence of unusual LRR motifs within the central portion. However, the Asparagine networks (N in LxxLxLxxNxL or LxxLxLxxNxxL) within TLR1 family members' central domains are absent. The Asparagine network supports the stability of the horseshoe-shaped configuration of TLR molecules [83].

**Figure 3:**
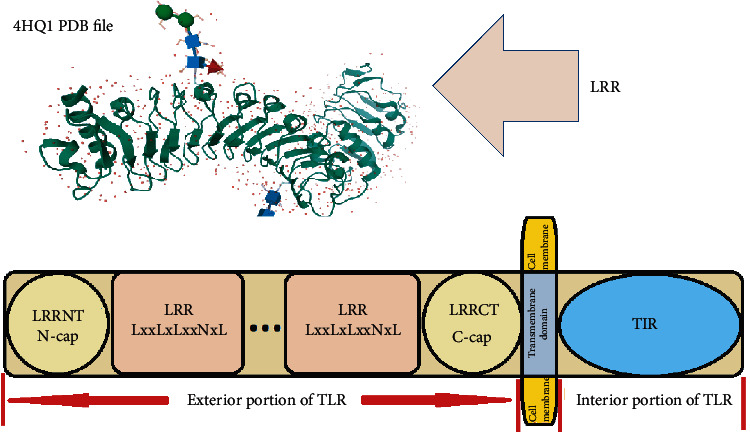
The structure of TLR and the related domains. The Ls in LxxLxLxxNxL depict hydrophobic core built of *β*-strands, and the N depicts the Asparagine network. The two-solenoid LRR structure shows the 3D structure of loops, helices (convex surface), and *β*-strands (concave surface) (4HQ1 PDB file) [87].

Hence, some distortions are seen in segments that miss Asparagine networks. The ligand-binding pockets in TLRs 1, 2, and 6 are located in the boundary region of central and CTDs. This characteristic explains why the bonds between internal protein pockets and LPS and/or lipoprotein ligands occur in TLR1 family members [83]. In TLR4 glycoprotein, one of the two MD-2 binding sites is situated in adjacent to the central and NTD's border. In contrast to nonviral TLRs (TLR1, TLR2, TLR4, and TLR6), the viral TLR biomolecules of 3, 5, 7, 8, and 9 encompass LRR domains with continuous Asparagine network and similar *β*-sheet angles. This property explains why the external protein pockets in viral TLRs bind to hydrophilic ligands of nucleic acids [83]. Furthermore, the CTD of the HSC section is connected to the NTD of the VS portion via ascending loops. In contrast, the CTD of the VS portion is joined to the NTD of the HSC portion through descending loops [79]. The N- and CTDs of the solenoid structure of LRR domain are capped by N-cap/LRRNT and C-cap/LRRCT, respectively. Both caps are armed by an even number of cysteine residues consisting of two, four, and six cysteines. The caps protect the hydrophobic core of the hydrophobic consensus sequences of the LRR motifs. The LRRs are usually flanked by their LRRNT and LRRCT within membrane proteins (Figure 3) [79, 83].

The ectodomain of a TLR molecule may involve 16-28 LRR different motifs [86]. The TLRs are categorized into different subfamilies following the number of their constitutional LRR domains and the motifs of two clusters belonging to cysteine amino acids located at the adjacent of the LRRs [86].

The LRR motifs are activated through their direct contact with the related ligands such as DAMPs, MAMPs, PAMPs, and XAMPs [8, 24, 28, 79]. Indeed, the TLR ligands are divided into three types of endogenous, microbial, and synthetic (agonists) ligands [88]. All the ligands of TLRs are shown in Table 1. Hence, binding the ligands to their specific binding sites on LRR domains of TLR glycoproteins leads to spatial changes in TLR dimer configurations. This feature results in changes in the spatial orientation of the cytosolic section of TIR domains. The consequence of these alternations is TIR adaptors activation, which may lead to produce intracellular signaling exchanges [89].

## 4. Toll/Interleukin-1 Receptor (IL-1R) (TIR): A Well-Known Superfamily

**Figure 4:**
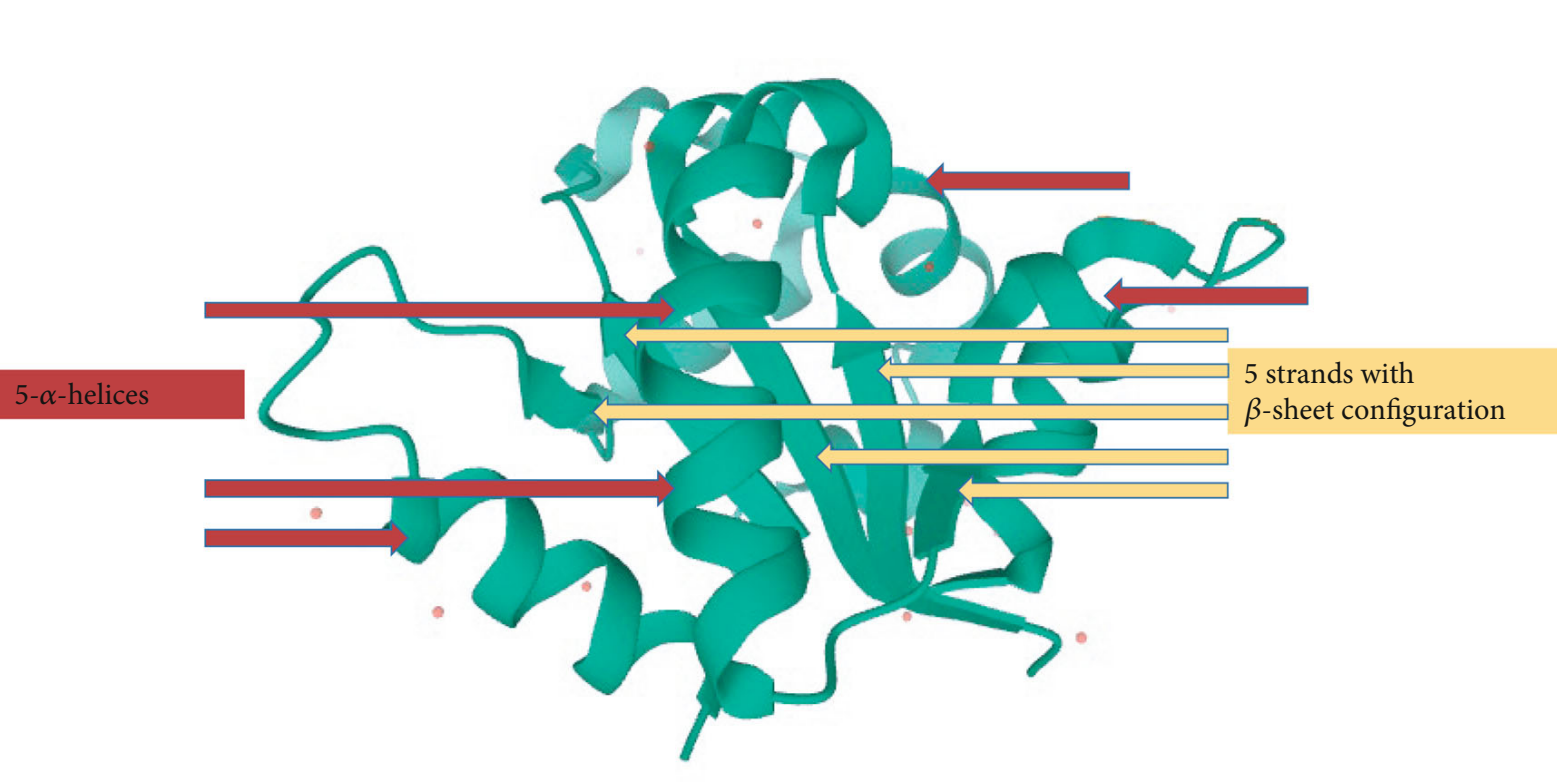
The structure of the TIR domain of TLR6 (4OM7 PDB file) [93].

The superfamily of TIR domain—as the cytoplasmic segment and signaling pathways initiator of TLR biosensors—is composed of a conserved structure involving five parallel strands with *β*-sheet configuration (*β*A-*β*E) within the core center enclosed by five *α*-helices (*α*A-*α*E) on either side, and eight loops bind altogether. These loops are named after their connection to the strands with their related secondary structure [6, 37, 90–92], e.g., CD loop. The CD loop binds the *α*C helix to the *β*D strand (Figure 4) [93].

The interleukin-1 (IL-1) receptors (IL-1Rs) as well as TLRs possess TIR domains in their structures; hence, they build together a superfamily [6]. Both TLR and IL-1R act as alarm receptors, while the ligands play their role as alarm mediators. Typically, the TLR signal transduction is triggered by MAMPs/PAMPs, DAMPs, and XAMPs, whereas the IL-1R signal transduction is occurred via different types of cytokines [94, 95]. The IL-1R family comprises of ten members pertaining to type I transmembrane proteins with the same architectural structures. They are three sectional structures composed of (i) an extracellular segment built up of three Ig-like domains known as D1, D2, and D3 on the N-terminus, (ii) a transmembrane domain, and (iii) an intracellular segment. The extracellular section of IL-1Rs is designed to detect the related ligands and binding them, whereas the intracellular fragment acts as the signaling pathway initiator [94].

The IL-1Rs act as receptors of the mediators which interact with endogenous alarm molecules. The IL-1R family members are versatile in functions and structures. The IL-1Rs are categorized into four groups including (i) accessory protein group (IL-1R3 and IL-1R7), (ii) ligand binding group (IL-1R1, IL-1R2, IL-1R4, IL-1R5, and IL-1R6), (iii) negative regulators group (IL-1R2, IL-1R8, and IL-1R18BP), and (iv) unknown IL-1R-like functional group (IL-1R9 and IL-1R10) [94]. Following previous bioinformatic and computational studies, the TIR domains encompass three conserved motifs, including box 1, box 2, and box 3 [89, 92, 94]. The boxes 1 (D-K-YDAF-SY) and 3 (-FWKx-) are conserved in TIR domains belonging to superfamily (TLRs and IL-1Rs) and adaptor protein of MyD88. The box 2 (GYKLCI-RD-PG) is conserved in TLRs and IL-1R, separately; in other words, the conserved sequences are specific to each group, respectively, and not in both of TLRs and IL-1Rs as an entire superfamily [89, 91, 94].

The previous bioinformatic and computational investigations reveal that TLR family members' TIR domains have up to ≥50% sequence similarity. However, a similarity with 87% has been reported for TIR domains between TLR1 and TLR6 [7, 90, 92]. TLRs initiate to dimerize in the presence of the related ligands, because dimerization may lead to some changes in TIR domains' spatial configurations. The TIR domains are present in both of TLR molecules and TLR-related signaling adaptors [6, 96]. The conformational changes in TLRs and adaptors TIR domains provide homotypic interactions between the TIR CTDs of the TLRs and the related signaling adaptors. However, no specific binding site is detected in TIR domains of TLR and signaling adaptors [6, 96]. Hence, the signaling adaptors mediate between TLRs and downstream kinases to convert ligand-TLR physical activities into the intracellular signals. This process results in the activation of nuclear factor B (NF-*κ*B), AP-1, IRFs molecules (important transcription factors), and expression of immune responses of proinflammatory and IFNs [6, 28, 95].

**Figure 5:**
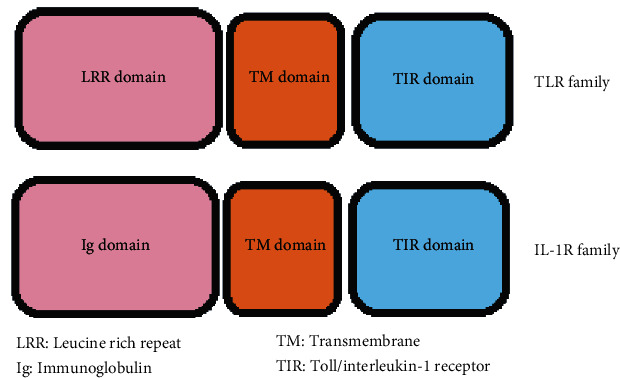
A comparison between TLR and IL-R families. Their difference is related to the LRR and Ig domains.

The binding of ligands to either IL-1Rs or TLRs leads to function of different signaling adaptors (depending on TLR glycoproteins), e.g., B-cell adaptor for phosphoinositide (BCAP), MyD88 adaptor-like (MAL)/TIR domain-containing Adapter molecule (TIRAP), MyD88, Sterile *α*-containing and Armadillo motif-containing protein (SARM), SLP65/76 and Csk-interacting membrane protein (SCIMP), TIR-domain containing adaptor-inducing INF-*β*- (TRIF-) related adaptor molecule (TRAM)/TLR Adaptor Molecule2 (TICAM2), and TRIF/TLR Adaptor Molecule1 (TICAM1) in the cytoplasmic section of the TIR domain. Activation of these adaptors is the cornerstone of TIR-TIR interactions [6, 7, 25, 41, 95]. The loop which binds the *β*B strand (second *β* strand) to the *α*B helix (second *α*-helix) bears the conserved motif of box 2. This bridge loop—known as the BB loop in TIR domains—is responsible for direct interactions between the adjacent TIR domains [89]. The TIR domains normally involve 125 to 200 residues and are usually connected with LRR and Ig domains (Figure 5).

TIR domains are identified in animals (mammals), bacteria, archaea, fungi, and plants. These domains interact with a wide range of proteins (with or without TIRs). The TIR-TIR interaction is known as homotypic interaction and the TIR-non-TIR interaction is known as heterotypic interaction [91, 92, 95]. All in all, a versatile of TIR domain structures are verified in which three main TIR-TIR interactions including A, R, and S face with specific signaling pathways occur [94]. The A face makes the TIR domain oligomerization easier in the MyD88 adaptor. The R face contributes to receptor TIR domain oligomerization. These domains have a pivotal role in signaling specificity. The S face modulates and regulates the binding between the receptor TIR domain and the adaptor TIR domain [94]. According to the information above, we are going to discuss the molecular and structural biology of TLRs.

## 5. General Mechanisms of TLR Biosynthesis and Trafficking

Each organism depending on its phylogenetic characteristics employs a limited number of TLRs. For instance, humans recruit 10 TLRs biosynthesized by their ER systems. The synthesized TLR molecules then continue their completion process of glycosylation. Then, TLR's trafficking process is completed by its transmission from ER into the *cis*-Golgi apparatus [41, 42, 63, 97, 98]. TLRs are synthesized within the ER because of the presence of pivotal chaperones including Unc-93 homolog B1 (Unc93B1-a polytopic (12 segmented) transmembrane protein), gp96 (Hsp90*β*1), and PRAT4A (CNPY3), which contribute to spatial folding and configuration of the cell membrane and endosomal TLRs. Gp96, as a member of the Hsp90 family, participates in spatial folding conformation and the activities of different proteins such as TLR glycoproteins [41, 42, 63, 99–103].

The absence of gp96 and PRAT4A chaperones within the associated cells may lead to incomplete folding of related TLRs' structures and results in the inactivation of TLR glycoproteins. However, the TLR3 is independent from gp96 and PRAT4A chaperones, and the absence of these chaperones does not affect its activities and functions [63, 104–106]. The presence of PRAT4A molecule is vital for the expression of cell surface TLRs of 1, 2, 4, and 5. Moreover, the PRAT4A protein contributes to the induction of TLRs 7 and 9 signaling pathways [42, 99, 107]. The 12-segmented transmembrane chaperone of Unc93B1 is the main protein that mediates nucleic acid-sensing endosomal TLRs. Besides, the Unc93B1 chaperone has a key role in the expression of TLR5 as the flagellin sensing cell membrane TLR [63, 108]. This chaperone makes the folding of endosomal TLR molecules easy through binding them within the ER space. The binding of Unc93B1 to the related TLR glycoprotein stabilizes the structure of associated TLR within the ER. Unc93B1 chaperone transfers nucleic acid-sensing endosomal TLRs from ER to destined endolysosomes [42, 109].

In the absence of Unc93B1, the nucleic acid-sensing endosomal TLRs cannot be internalized within the endolysosomes, and they should stay within the ER [42, 109]. Furthermore, Unc93B1 facilitates the cleavage of TLR molecules; a feature that helps TLRs to detect their ligands to bind them and initiation of the related signaling pathways. The Unc93B protein binds to TLR glycoproteins through some acidic amino acid residues which are located in the juxtamembrane region, a zone between the LRR and the transmembrane domains [42, 110]. The Unc93B1 chaperone contributes directly to the TLR secretion pathway. In other words, the Unc93B1 governs the packaging process of TLR molecules, which is achieved via the budding mechanism from the ER network in the form of coat protein complex II (COPII) vesicles. During the Golgi apparatus post sorting processes, the Unc93B1 protein is bound to TLR molecules [25, 41, 63, 111].

The dissemination and trafficking processes of TLRs from the Golgi apparatus differ for different TLR molecules. The TLRs of 7 and 11-13 located within the *cis*-Golgi apparatus are directly transferred into the endolysosomes. The trafficking adaptor—adaptor protein complex 4 (AP-4)—possibly mediates these interactions. In contrast, the TLR9 protein is transferred within several steps. First, TLR9 is transmitted into the cell membrane, and then, the TLR9 together with Unc93B1-AP-2 is delivered to endolysosomes [25, 41, 63, 111]. According to recent studies [63, 100], the TLR7 molecule remains together with the Unc93B1 chaperone after the delivery process to the endolysosomes. This process may lead to TLR7 signaling inhibition. It seems that the posttraffic activity of Unc93B1 is associated with TLR7 to internalize the TLR7 glycoprotein into multivesicular bodies to terminate the signaling pathway of TLR7 [63, 99, 100, 109].

Simultaneously, the Unc93B1 in parallel with TLR9 internalization will be disconnected and allows the TLR9 to bind with its ligand to induce the related signaling pathways. The signaling activities associated with TLRs 7 and 9 are directly balanced by Unc93B1 [63, 99, 100, 109]. By the localization of endolysosomal TLRs consisting TLR3 and TLRs of 7-9 within their positions, some changes should be done to be activated. Therefore, some cathepsins (e.g., B, S, L, H, and K) and asparaginyl endopeptidase are recruited to cleave a portion of LRR domains belonging to TLR3 and TLRs of 7-9. The cleavage of LRR domains makes the TLR glycoproteins functional (activated by dimerization) [63, 99, 109, 112].

Although the noncleaved TLR3 and TLRs of 7-9 can be identified and bind to their specific ligands, they cannot be dimerized; hence, their activation and induction of the related signaling pathways depend on TLRs cleavage in their ectodomains (LRRs) [63, 109, 113–115]. Hence, the endosomal TLRs are needed to be cleaved to be activated and initiating signaling transduction, while this process is not necessary for cell surface TLRs [63]. The general process of TLR dimerization is done via juxtamembrane sequences, which are located upon the CTD of the two adjacent ectodomains. The juxtamembrane sections involve a stabilized antiparallel *β*-sheet [41]. The stable condition appears by the formation of two disulfide bonds. The juxtamembrane sequences are bridged to the transmembrane helix through a very tense linker made of ~three amino acids [41]. The ectodomains act as dimerization inhibitors, while by binding of ligands to the TLRs reduces this property. Therefore, both of the juxtamembrane and transmembrane domains tend to combine and dimerization [41, 116].

### 5.1. TLR1

TLR1 is a cell membrane molecule activated by coreceptors of TLR2, TLR6, and 10 [8, 23, 28, 71, 86]. A close similarity is identified between the TLR glycoproteins of 1, 6, and 10 sequences [27]. The ligand-specific recognition and signaling pathway initiation in TLR1 begin by the process of heterodimerization between TLR1 and the related coreceptors of TLR2, TLR6, and TLR10 [23, 28, 71, 86]. The gene cluster of *TLR6-TLR1-TLR10* encodes the TLR molecules of 6, 1, and 10 and maps to human genomic chromosome 4p14. The human chromosome 4 also bears *TLR2* and *TLR3* genes (Table 1) [23, 28, 47, 117, 118]. The crystal structure of TLR1-TLR2 heterodimer indicates the horseshoe configuration in their LRR motifs. The horseshoe-like structure of ectodomains in each portion of TLR1-TLR2 heterodimer builds an “m” letter-like TLR1-TLR2 heterodimer which is bound to the related ligands such as microbial triacyl lipopeptides (MAMPs/PAMPs) in *Mycoplasma* spp., Gram-negative bacteria, and the agonist of Pam_3_CSK_4_ (XAMP). The “m” letter-like configuration of TLR1-TLR2 heterodimer is the result of stretched out NTDs and merging the CTDs in the central section of the TLR1 and TLR2 molecules [8, 26, 28, 71, 86, 119–121].

The bacterial membrane protein family, e.g., lipopeptides or lipoproteins in Gram-negative bacteria encompass three chains of lipid. Two chains of three are bound to the glycerol molecule. The glycerol is covalently joined to a sulfur atom [122]. This atom (sulfur) belongs to the conserved cysteine residue, which is located on the NTD. These two chains of lipids are known as ester-bound lipid chains. The third lipid chain—known as the amide-bound lipid chain—is bound to the NH2 group (situated on the NTD) via an amide bond. The glycerol and amino acid residues upon NTDs of lipopeptides bind to amino acid residues of TLR1-TLR2 via enormous hydrogen bonds [122]. The TLR1 and TLR2 have their own tight and tense hydrophobic channels and pockets. Each TLR has its own specific characteristics. When the TLR1 and TLR2 are dimerized, their spatial configurations change and they build a long mutual hydrophobic pocket [8, 119, 120, 122]. By binding of Pam_3_CSK_4_ as an agonist to TLR1-TLR2 mutual hydrophobic pocket, the third lipid chain (amide-bound lipid chain) of Pam_3_CSK_4_ connects with the short hydrophobic pocket of TLR1. In contrast, the left two chains (ester-bound lipid chains) of Pam_3_CSK_4_ bind to the large hydrophobic pocket of TLR2. This process guarantees the stability of the TLR1-TLR2 heterodimer configuration [8, 119, 120, 122].

The kernel of the interface zone between TLR1 and TLR2 in TLR1-TLR2 heterodimer is built of many hydrophobic residues in which this core is enfolded by hydrophilic amino acids constructing hydrogen and ionic bindings [8, 119, 120, 122]. Previous studies show that the large hydrophobic pocket of TLR2 is located at the adjacent of the border region between CTD and central domain which expands into a small internal hydrophobic pocket made of four LRRs, including ninth to twelfth (LRR9, LRR10, LRR11, and LRR12) [122]. The ligand-TLR1-TLR2 complex induces TIRAP and Myeloid differentiation primary response gene 88 (MyD88) signaling adaptors, which belong to downstream signaling transduction. The result is the expression of NF-*κ*B, which ends with production of inflammatory cytokines [26, 121, 123].

### 5.2. TLR2

The *TLR2* gene is located on the 4^th^ chromosomal genome; it maps to 4q32 [23, 28, 47]. TLR2 is a flexible glycoprotein and has the capability of dimerization with its coreceptors such as TLR1, TLR2, TLR6, and TLR10. Although it is suggested that TLR2 molecules can build TLR2-TLR2 homodimers, no experimental observation has been reported up to date [8, 27, 86, 121, 124]. The specific characteristics of TLR2 enable this molecule to identify a versatile of MAMPs/PAMPs originated from a wide range of microorganisms involving bacteria (Gram-positive bacteria, Gram-negative bacteria, and Mycobacteria), parasites (e.g., *Trypanosoma cruzi* and *Plasmodium falciparum*), fungi, and viruses [23, 26, 28, 71, 86, 121]. As aforementioned, the TLR1-TLR2 heterodimers are able to detect triacylated lipopeptides belonging to Gram-negative bacteria and *Mycoplasma* spp., while the TLR2-TLR6 heterodimers identify the diacylated lipopeptides of Gram-positive bacteria and *Mycoplasma* spp. [98, 121]. The TLR2 molecules encompass a high affinity to the related ligands with low concentration [63].

The sTLR2 molecules which are produced through the ectodomain shedding or protease cleavage may lead to the appearance of ≥6 separate polypeptides of sTLR2 [21, 125]. The sTLR2 polypeptides can be involved in dimerization with cell surface of TLR2. Besides, the sTLR2 can be recognized as an important competitor for cellular TLR2 molecules in the presence of microbial ligands [10, 11]. TLR2 employs the MyD88 as a downstream adaptor to activate signaling transduction. The BB loop plays a pivotal role in the binding of the TLR2 TIR domain to signaling adaptors. Moreover, the BB loop exterior amino acid residues, e.g., Pococorante site, contribute to employ MyD88 adaptor for initiating signaling transduction via TLR2 [121, 123]. The BB loop situated in TLR2 TIR domain is flexible which is confirmed through computational modeling. This BB loop makes TLR2 flexible in heterodimerization with the coreceptors of TLR1, TLR2, and TLR6 [86, 121]. Furthermore, this loop supports the TLR2 interactions with a diversity of the related ligands, because the BB loop is able to have different spatial configurations in versatile conditions in association with different complexes including the TLR1-TLR2, TLR2-TLR6 heterodimers, and TLR2-TLR2 homodimers and their specific ligands. The function of TLR2-TLR10 heterodimers is unknown [86, 121]. Qiu et al. have shown that two amino acid residues, situated on the exterior section of the BB loop on TIR domain, have a crucial role in the induction of signaling transduction regarding TLR1-TLR2 and TLR2-TLR6 heterodimers as well as the homodimer of TLR2-TLR2 [121].

In other words, the exterior amino acid residues of BB loop have a crucial role in using the MyD88 signaling pathway by the TLR2 molecules. Besides, there is a 681P as an interior amino acid residue situated within the BB loop. The 681P is able to determine which signaling transduction belongs to either the heterodimers of TLR1-TLR2 and TLR2-TLR6 or the homodimer of TLR2-TLR2 [121]. The single internal amino acid residue of the BB loop (681P) and the two amino acid residues situated out of the BB loop bilaterally are conserved in the TIR domain of all TLR molecules except TLR3 [121]. The TLR2 molecules activate the MyD88 downstream adaptors, and in follow, the NF-*κ*B will be expressed, which results in the expression of inflammatory cytokines as the final products. However, it seems that the TLR2-TLR10 heterodimer induces a signaling transduction rather than the MyD88 pathway [26, 86, 123, 124]. The process of TLR2 heterodimerization is performed by the help of coreceptor molecules [86]. This property of TLR2 shows its high ability to interact with different ligands and molecules via a wide range of mechanisms (e.g., through the BB loop) [86, 121].

TLR1-TLR2 heterodimers need CD14/GD1a, CD14/CD36, and CD14/vitronectin-integrin *β*3 as coreceptors for binding to ligands of heat-labile enterotoxins (b subunit), mycobacterial lipomannan and lipoarabinomannan, and bacterial triacyl lipopeptides, respectively. On the other hand, the TLR2-TLR6 heterodimers need different coreceptors to bind to their specific ligands. RP105 and CD14/CD36, CD14/CD36/MBL, CD14/dectin-1, and CD14 are, respectively, known as TLR2-TLR6 coreceptors to bind to the related ligands such as mycoplasma diacyl lipopeptides, Gram-positive lipoteichoic acid, fungal zymosan, and viral glycoprotein B [86]. CD14 is an LRR glycoprotein consisting of 375 amino acids, which can be detected in blood as a soluble molecule or located in membrane protein on myeloid cells in the form of glycophosphatidylinositolated protein. The functional form of CD14 is CD14-CD14 homodimer, with a horseshoe-like structure resembling the ectodomain portion of TLR molecules. Each CD14 encompasses a hydrophobic pocket on its NTD. This hydrophobic pocket forms a ligand-binding site. The CD14 coreceptor can bind to a wide range of ligands, including Poly(I : C), peptidoglycan, Pam3CsK4, LPS, lipoteichoic acid, double-stranded DNA (dsDNA), and CpGDNA; thus, the CD14 interacts with TLR molecules of 2-4 and 7-9 [63, 126–132].

It is reported that the sTLR2 halts TLR2 to bind to its own coreceptor of CD14. This process may lead to inhibit the inflammation responses modulated by TLR2 [13, 21]. Furthermore, the coreceptor of CD36, which is known as a double-spanning membrane glycoprotein, consisted of 472 amino acids. CD36 as a member of scavenger receptor type B family binds to some heterodimers, e.g., TLR2-TLR6 and TLR4-TLR6, to build TLR2-TLR6-CD36 and TLR4-TLR6-CD36 complexes. The CD36 molecule can promote immune responses against some ligands of TLR2-TLR6 and mediates inflammatory responses against some DAMPs (endogenous ligands) of TLR4-TLR6 [130, 133–137].

### 5.3. TLR3

The endolysosomal *TLR3* gene maps to 4q35 on human chromosome 4 [23, 28, 47]. TLR3 possesses a large size horseshoe-like ectodomain, which is consisted of 23 LRR motifs. By the process of TLR3 homodimerization, the N-terminal portion of ectodomains stretches outward and in the opposed direction which may lead to form an “m” letter-like configuration in the TLR3-TLR3 homodimer. In consequence, a considerable space is provided for determining the negatively charged molecule of viral double-stranded RNA (dsRNA) to binding with. The TLR3 homodimers are able to identify some molecules of single-stranded RNA (ssRNA). Sometimes along with viral infections, replication of the positive sense of ssRNA molecule may be achieved via a dsRNA intermediate; this mechanism allows the TLR3 homodimers to recognize this kind of viral ssRNA molecules. Each TLR3 molecule bears two RNA binding sites situated on each monomer's N- and CTD solenoids [8, 23, 26, 42, 120, 122, 138, 139].

The ligand molecule of viral dsRNA attaches to two different binding sites situated upon the N- and CTDs of the two opposite ends of the horseshoe-like structure. The NTD-containing binding site portion consists of LRRs 1-3 and LRRNT, while the CTD involving the binding site portion is comprised of LRRs 19-21. These domains are located on the lateral side of the convex surface of the TLR3 ectodomain. The ligand attachment to the TLR3 binding sites stabilizes the spatial configuration of TLR3 molecules to construct homodimers via their CTDs [8, 26, 120, 140]. The TLR3 molecules encompass a high affinity to the related ligands with low concentration [63]. A TLR3-TLR3 homodimer binds to those viral dsRNA ligands, which are longer than 40 bp. This means that the horseshoe-like space covers at least ~two helical turns of RNA. The previous studies show that the backbone of the dsRNA molecule containing phosphates and sugars has a pivotal role in attachment to the TLR3-TLR3 binding sites, whereas the nucleic acid bases have the least contribution. The suitable pH for TLR3-TLR3-ligand complex formation is 6.0, because of the presence of several histidines which bind to phosphate molecules of the viral dsRNA backbone [122, 140]. CD14 and Mex3B are the coreceptors of TLR3, which participate in the formation of TLR3-TLR3-CD14-dsRNA and TLR3-TLR3-Mex3B-dsRNA complexes [42, 141].

In contrast to other TLR molecules, the TLR3 recruits the TRIF signaling pathway. This signaling transduction results in the expression of NF-*κ*B and IRF3, which may lead to the production of two different types of molecules, including inflammatory cytokines and type I IFN, respectively [26, 123]. The reason that why the TLR3 employs the TRIF signaling pathway refers to the structure of the BB loop in the TIR domain. As aforementioned, the conserved amino acid residues in the inner and outside of the BB loop are similar in all TLR molecules, except TLR3. This characteristic of the BB loop of TLR3 TIR explains these cornerstone differences [121].

### 5.4. TLR4

TLR4 glycoprotein with specific characteristics and a wide range of functions is known as a critical TLR molecule among others. TLR4 biomolecules depending on their biological properties can be detected upon cell surfaces and/or within the cells expressed by endolysosomes. TLR4 molecules bind to a versatile of ligands, including MAMPs/PAMPS (e.g., Gram-negative bacterial lipopolysaccharide (LPS)), DAMPs, and XAMPs [8, 26, 98, 122, 123].

Compared with TLRs 2, 3, 5, and 9, the TLR4 needs a higher concentration of the related ligands to have high affinity [63]. *TLR4* gene maps to 9q32-33, which is located on chromosome 9 of human genome [23, 28, 47]. TLR4 is known as the first identified TLR in mammals [142]. The functional molecules of TLR4 act in the form of TLR4-TLR4 homodimers. TLR4 binds to its specific ligands with the help of the MD-2 coreceptor molecule. The MD-2 molecule binds to the extracellular domain of TLR4 to be expressed upon the cell membranes. This process results in the binding of TLR4-MD-2 to the ligand of LPS. MD-2 is a soluble glycoprotein with ~18 kDa weight, containing 160 amino acid residues with a sandwich structure of two antiparallel *β*-sheets which lack disulfide bond. The absence of disulfide bond allows MD-2 (in TLR4-MD-2 complex) to change the antiparallel *β*-sheets spatial configuration to build a bulky internal pocket. This new configuration provides a suitable condition for TLR4-MD-2 complex to bind to LPS. The LPS encompasses six lipid chains containing 12-14 C atoms, in which five of them will be placed within the hydrophobic pocket of MD-2. These five lipid chains fill up the space of the pocket, which stabilizes the bond. The empty pocket of MD-2 may lead to the destabilization of the bond. The sixth lipid chain binds to the TLR4 [26, 63, 83, 120, 122, 130].

So as aforementioned, TLR4 homodimer makes a complex with two coreceptors of MD-2 in the form of two copies of TLR4-MD-2. Then, they will bind to the ligands of LPS to produce two copies of TLR4-MD-2-LPS complex. Due to this knowledge, the MD-2 has a vital role in the homodimerization of TLR4 glycoproteins and ligand attachment. Therefore, the coreceptor of MD-2 bears two specific binding sites comprised of dimerization interfaces for attaching to TLR4 [27, 120, 122, 130]. Indeed, LPS is a glycolipid consisted of a lipid A (a negative charged hydrophobic structure) and a carbohydrate chain (which is long and branched). The presence of phosphate groups in lipid A structure supports the negative charge and hydrogen binding interactions. The phosphate groups (1′ and 4′) contribute to bind to the lysine and arginine amino acid residues of the TLR4 and MD-2 molecules. The appearance of ionic and hydrogen bonds between phosphate groups and amino acid residues of lysines and arginines supports the process of TLR4 homodimerization [37, 120, 122]. Through the formation of TLR4-MD-2 complexes, the soluble plasma proteins of LPS binding protein (LBP) bind to LPS. In follow, the LRR motif-containing protein of CD14 (which is attached to a glycosylphosphatidylinositol) connects to the LBP section of the LBP-LPS complex and allows the LPS ligand to bind to the TLR4-MD-2 complex. The LBP is known as an acute-phase protein, which consisted of 481 amino acid residues. It has a significant tendency to bind to Gram-negative bacterial LPS. Moreover, the LBP binds to lipopeptides, peptidoglycan, and lipoteichoic acid too. Hence, all of these ligands can be transmitted by LBP to the CD14 coreceptor. The LBP cooperates with CD14 in association with functional TLR1-TLR2, TLR2-TLR6, and TLR4-TLR4 [8, 26, 63, 122, 127, 130, 143–146].

The sTLRs of 2 and 4 are produced in presence of the related ligands like lipopolysaccharide (LPS) via the both situations of *in vivo* and *in vitro*. Moreover, the concentration of sTLR4 molecules increases in patients with bacterial infectious diseases rather than those patients infected by viruses. Indeed, the sTLRs of 2 and 4 form an effective negative regulatory construction in the first-line position [11]. The sTLR4 is able to bind to the MD-2 coreceptor to form a complex to prevent any interaction between the cell surface molecule of TLR4 and the related microbial ligands [11, 21, 147].

RP105—a type I receptor—is another LRR protein that acts as a coreceptor with functional TLR4 to identify the LPS in B cells. RP105 possesses an ectodomain like TLR4, but not like a TIR CTD. The MD-1, a member of the ML family of the lipid-binding protein, binds to LPS. The MD-2 (as discussed before) is another member of the ML family of lipid-binding protein bind to LPS. Although the MD-1 and MD-2 function on different surfaces, they both distinguish the same ligand of LPS. The RP105-MD-1 complex binds straightly to the TLR4-MD-2 complex and promotes the immune responses against LPS [37, 148]. TLR4 has a crucial role in different types of diseases [8]. The endosomal TLR4 resembling TLR3 activates the TRIF-dependent signaling pathway. However, the cell membrane TLR4 employs the TIRAP adaptor molecule to switch the MyD88-dependent pathway by the TIR domain. Hence, by the appearance of the TLR4-MD-2-LPS complex upon the cell membrane, the MyD88-dependent signaling pathway is activated. This signaling pathway results in the expression of the early phase of NF-*κ*B and the production of inflammatory cytokines as the final products. By the internalization of the TLR4-MD-2-LPS complex into an endosome (or lysosome), the TRIF-dependent signaling pathway is recruited. The activation of the TRIF-dependent pathway is switched by the TIR domain of endolysosomal TLR4, in which the TRAM adaptor is activated and leads to the expression of IRF3 and late phase NF-*κ*B. The activation of IRF3 ends to the expression of type I IFN, while the activation of late-phase NF-*κ*B leads to the expression of inflammatory cytokines [26, 28].

The Gram-negative uropathogenic bacteria including uropathogenic *Escherichia coli* (UPEC), uropathogenic *Klebsiella pneumoniae* (UPKP), uropathogenic *Proteus mirabilis* (UPPM), and uropathogenic *Pseudomonas aeruginosa* (UPPA) are effective MAMPs (encompassing bacterial LPS) which activate the TLR4 signaling pathways [23, 71, 123, 149–158].

### 5.5. TLR5

*TLR5* gene maps to 1q33.3 on genomic chromosome 1. TLR5 is known as bacterial flagellin sensing. TLR5 and TLR11 (in mice) are in association with each other. Both of them detect the uropathogenic bacterial flagellins [8, 23, 26, 28, 47, 120]. The flagellin core domains, including D1, D2, and D3 (FliC fragments), have a pivotal role in TLR5-flagellin bond. D1 domain—bearing three *α* helices (long structures) and a *β* hairpin—is significantly conserved and has a key role in assembling fliC subunits and their polymerization into a helical filament. Two helices of three in the D1 domain involve N- and the third helix involves the CTD of the flagellin. Now, we know that the D1 domain has a bold participation in binding and dimerization of TLR5 and acts as a bifunctional domain [8, 27, 37, 42, 120, 122].

On one hand, D1 binds to the exterior section of TLR5 ectodomain, from LRRNT to LRR9 in one of the TLR5 molecules in a TLR5-TLR5 homodimer, and on the other hand, D1 binds to the second TLR5 molecule in the same TLR5-TLR5 homodimer. In other words, the flagellin binds to the lateral sides of the TLR5 molecules. This interaction leads to the stabilization of the TLR5-TLR5 homodimer structure. However, in the absence of the ligands, the TLR5-TLR5 homodimers are seen; the homodimers are combined of two horseshoe-like ectodomains with the appearance of a letter m-like structure. The stability of the horseshoe-like structure of the TLR5 molecule depends on the rigidity of LRR folding and spatial configuration [8, 27, 37, 42, 120, 122]. The TLR5 molecules encompass a high affinity to the related ligands with low concentration [63].

The motile Gram-negative uropathogenic bacteria including UPEC, UPPM, and UPPA are effective MAMPs (encompassing flagellins) which activate the TLR5 signaling pathway [23, 71, 123, 149–158]. TLR5 molecules activate the MyD88-dependent signaling pathway, which leads to the expression of NF-*κ*B and, in consequence, results in the production of inflammatory cytokines [26].

### 5.6. TLR6

The *TLR6* gene is located on the gene cluster of *TLR6-TLR1-TLR10*, an operon that maps to 4p14 on chromosome 4 [28, 117]. TLR6 as a cell membrane molecule can be functional only in the form of TLR2-TLR6 and TLR4-TLR6 heterodimers. The transmembrane domains of TLRs 2 and 6 and TLRs 4 and 6 have the leading role in TLR2-TLR6 and TLR4-TLR6 heterodimerization. In the process of TLR4-TLR6 heterodimerization, the presence of coreceptor of CD36 is necessary; therefore, the functional TLR4-TLR6 is in the form of TLR4-TLR6-CD36 complex [8, 159–161]. The related MAMPs/PAMPs of TLR1-TLR2 and TLR2-TLR6 heterodimers differ from each other. The well-known MAMPs/PAMPs of TLR1-TLR2 are triacylated lipopeptides, while the well-known MAMPs/PAMPs of TLR2-TLR6 are diacylated lipopeptides [23, 26, 28, 71, 122]. As mentioned before, the triacylated lipopeptides encompass three lipid chains. Two of them (ester-bound lipid chains) bind to the TLR2 hydrophobic pocket, whereas the third one (the amide-bound lipid chain) binds to TLR1 hydrophobic channel. In contrast, the TLR6 molecule has no hydrophobic pocket. In other words, the hydrophobic channel in TLR6 is plugged by two huge phenylalanine amino acids, and the amide-bound lipid chain cannot be bound to TLR6. Hence, the ligand of TLR2-TLR-6 heterodimer is diacylated lipopeptides and not triacylated lipopeptides [26, 122]. The TLR6 together with its coreceptors including TLRs 2 and 4 activates the TIRAP adaptor, which results in the induction of the MyD88-dependent signaling pathway. This pathway activates the expression of NF-*κ*B in which may lead to the production of inflammatory cytokines [26].

### 5.7. TLR7

*TLR7* gene maps to Xp22.3 on the genomic X chromosome; TLR7 as well as TLR4, is known as endolysosomal and cell membrane TLR [23, 28, 47]. TLR7 resembling TLRs 3 and 9 is known as the nucleic acid-sensing molecule. However, the TLRs 3 and 9 are activated by dsRNA and dsDNA, respectively, while TLR7 is activated in the presence of viral ssRNA (e.g., HIV and influenza A virus). TLR7 is able to identify nucleotides and nucleosides related to intracellular pathogenic microorganisms. Besides, TLR7 is very close to TLR8; in other words, the homology and function of the TLRs 7 and 8 are very close to each other. Hence, both of them are activated by the purine-rich ssRNA molecules. The process of TLR7 dimerization occurs via a ligand-binding feature. Interestingly, the TLR7 biomolecules resembling TLR4 glycoproteins are activated in the presence of a high concentration of ligands [8, 23, 63, 140, 162–165].

The TLR7 molecule bears two binding sites to bind with its specific ligands. According to previous studies, the first binding site is conserved and binds to small ligands such as agonist R848 (a guanosine derivative), while the second binding site binds to ssRNA molecules. The second binding site in TLR7 promotes the function of the first binding site. The inactive horseshoe-like monomer molecule of TLR7 encompasses a Z-loop situated after LLR14 and before LRR15 [37, 63, 140, 162, 166–168].

The presence of ligand and attachment of small molecule of ligand to the first binding site of the TLR7 molecule induces the process of TLR7-TLR7 homodimerization and the letter m-like structure appears. The first binding site is situated at the adjacent of TLR7 dimerization interface; therefore, the first binding site activates the second binding site, to connect to the ssRNA. The first binding site has a strong tendency to guanosine and the attachment of the ssRNA molecule to the second binding site promotes the tendency of the first binding site for binding to guanosine. The ssRNA molecule which is known as the second binding site ligand should contain minimally three bases with a uridine-rich sequence. The presence of uridine nucleotides in ssRNA represents the ssRNA molecule as a monomer molecule to the second binding site [37, 63, 140, 162, 166–168]. As guanosine and ssRNA have a synergistic effect on TLR7 dimerization and activation, it seems that the TLR7 molecules are activated by degraded sequences of RNAs and DNAs rather than the ssRNA molecules [140, 167]. The molecule of TLR7 activates the MyD88-dependent signaling pathway, which leads to the expression of IRF5 and IRF7 molecules and induction of inflammatory cytokines and type I IFN production, respectively [26, 169].

### 5.8. TLR8

TLR8 resembling TLR7 is an ssRNA sensing TLR which have close properties to TLR7. *TLR8* gene like the *TLR7* gene maps to Xp22 where is located on the genomic X chromosome [23, 28, 47]. *In toto*, the phylogenetic studies confirm that TLRs 7-9 are proteins comprised of extracellular ectodomains, in which each of them is made of more than 800 amino acids. Although these TLRs have their own structures and activities, they are categorized into the same subfamily [170]. TLR8 like TLR7 bears two binding sites. The first binding site (involving LRRs 11-14 and 16-18) is conserved and binds to small ligands including uridine, whereas the second binding site binds to guanosine-rich ssRNA. The molecule of uridine binds to the first binding site through hydrogen bonds. The second binding site recruits the concave surface covering LRRs 10-13 and the 469-474 residues of the Z-loop region. TLR8 similar to TLR7 is the right receptor for degraded products obtaining from RNAs and DNAs. Therefore, it seems that both of TLRs 7 and 8 are nucleoside biosensors rather than ssRNAs. In contrast to TLR7, TLR8 senses guanosine-rich ssRNA. Moreover, TLR8, like TLR7, has two binding sites. The first binding site is located at the adjacent of dimerization interface, while the second binding site is out of the dimerization interface region [140, 162, 165, 166, 168, 170].

Unlike TLR7, the TLR8 molecules are in the form of TLR8-TLR8 dimers, and through binding to their specific ligands, the spatial configuration of TLR8-TLR8 dimer changes. The horseshoe-like external ectodomain of TLR8 involves the highest LRR domains (26 LRR motifs) among identified TLRs. TLR8 resembling TLRs 7 and 9 possesses an insertion region, which is known as Z-loop. This Z-loop is made up of ~40 amino acid residues (442-481) and is located between the LRRs of 14 and 15. At the end of Z-loop, a single turn of an *α*-helix is stabilized by a disulfide bond. The disulfide bond which occurs between two amino acids of cysteine 479 and cysteine 509 within the LRR16 is conserved in the subfamily of TLRs 7-9. Now, we know that the Z-loop has a crucial role in the identification of ssRNA ligand in both TLRs 7 and 8 [166, 168, 170]. TLR8 activates the MyD88-dependent signaling pathway, in which induces IRF5 and IRF7 molecules and the expression of inflammatory cytokines and type I IFN, respectively [169].

### 5.9. TLR9

*TLR9* gene maps to 3p21.3 situated on genomic chromosome 3 [23, 28, 47]. TLR9 belongs to the TLR9 subfamily (including TLRs 7-9) and senses those ssDNA molecules which encompass unmethylated cytosine-phosphate-guanine (CpG) motifs. The unliganded TLR9-TLR9 homodimers exist (like TLR8 and unlike TLR7 glycoproteins), and the presence of the related ligand may lead to a change in the spatial configuration of the homodimers. Each horseshoe-like extracellular ectodomain of TLR9 consists of 25 LRRs [8, 98, 120, 140, 166, 170–172].

Resembling TLRs 7 and 8, the TLR9 bears a Z-loop between the LRRs of 14 and 15. But in contrast to TLRs 7 and 8, the Z-loop does not contribute to determine the ligand molecule of CpG containing DNA. The LRRNT of the TLR9 is free and positively charged. The CpG-DNA ligand binds symmetrically to each TLR9-TLR9 homodimer, which may lead to the occurrence of a stoichiometric complex of 2 : 2. The ligand occupies the N-terminal of the LRR cluster, including LRRNT to LRR10 from one protomer and the C-terminal LRR cluster comprising LRRs 20-22 from the other protomer [8, 98, 120, 140, 166, 170–172].

TLR9 is the only hTLR which is capable to detect pathogenic DNAs of CpG-DNA within the endolysosomal structures. TLR9 has the ability of movement from ER into the other cellular structures such as endosomes, lysosomes, and endolysosomes which encompass CpG-DNA molecules [29, 173, 174]. The efficacy of the signaling pathway pertaining to TLR9 against the pathogenic bacterial molecules of CpG-DNA depends directly on the CpG-DNA concentration, content of CG dinucleotides, microbial species (*Ps. aeruginosa*>*K.pneumoniae*>*E. coli*), and the cytosolic presence of CpG-DNA [29, 175, 176]. TLR9 recruits the MyD88-dependent signaling pathway to activate the IRFs of 5 and 7, which may lead to the expression of inflammatory cytokines and type I IFN molecules, respectively [26, 169].

### 5.10. TLR10

*TLR10* gene mapped to 4p14 on chromosome 4 and located within the gene cluster of *TLR6-TLR1-TLR10* [23, 28, 47, 117]. TLR10 molecules dimerize with TLRs 1 and 2 as heterodimers and with TLR10 as homodimers. TLR10 has phylogenetic similarities with TLRs 1 and 6. Viral glycoproteins and dsRNA molecules activate the TLR10 as a cell membrane and endolysosomal molecule in humans. The *TLR10* in mice is known as a pseudogene [6, 23, 117, 118].

## 6. Conclusion

In recent years, the application of TLR agonists as distinctive immunomodulator agents represents a new option for induction of immune responses and effective vaccine adjuvants [177]. TLRs are versatile and invaluable biomolecules which have their own molecular and structural biology. Depending on their functions and activities, they have their unique characteristics and properties. The occurrence of heterodimers, homodimers, bond with different coreceptor(s), and a wide range of ligands indicates that these biomolecules are the core of immune and non-immune systems. Effective knowledge about TLRs provides us a brilliant promise to recognize these immune glycoproteins as effective immunogenetic targets for ligand-drug discovery strategies to establish new therapeutics in the fields of infectious diseases, cancers, and autoimmune diseases.

As the reported results show, the TLR agonists as a new class of immunomodulators offer an effective protection with a significant long-activity against a wide range of MAMPs/MAMPs through promoting the innate immune system activities. Nowadays, a wide range of TLR agonists comprising CU-CPT22, SMU-Z1, CU-T12-9, Pam_2_Cys (protects against influenza virus with long-term protectivity and secondary bacterial infections caused by *Streptococcus pneumoniae*) and Pam_3_CSK_4_ (TLR1/TLR2 stimulators), Pam_3_Cys (TLR2/TLR6 stimulator), polyinosinic : poly-cytidylic acid (Poly(I : C) a synthetic dsRNA molecule which activates TLR3 provides protection against viral infections [178]), CU-CPT4a (prevents binding of dsRNA to TLR3) [8, 177, 179–181], monophosphoryl lipid A (MPLA) (structurally resembles LPS and provides protection against viruses such as influenza virus; fungi like *Candida albicans*; Gram-negative bacteria, e.g., *Ps.aeruginosa*; and Gram-positive bacteria e.g., *Staphylococcus aureus* [182–184]. MPLA can act as an effective adjuvant in vaccines against malaria, HPV, and hepatitis B [185, 186]); TH1020 (inhibits TLR5 dimerization); and imiquimod (a synthetic imidazoquinoline) (TLR7 agonist and antagonist), and imidazoquinoline-based agents (TLR8 agonists and antagonists) are produced and some of them are approved by FDA for their use as vaccine adjuvants [8, 177, 179–181]. All in all, the ongoing studies provide us an interesting promise to use TLR agonists, antagonists, and vaccine adjuvants as effective immunomodulators and therapeutics for treating infectious and autoimmune diseases, cancers, and other inflammatory diseases and disorders.

## Figures and Tables

**Table 1:** Different characteristics of *TLR* genes and TLR proteins in human.

TLR member	TLR coreceptor	TLR family	Gene	TLR genes classification	TLR gene sequences	Gene position	TLR position	TLR active form	Ligands			Adaptors	References
									DAMP (endogenous)	MAMP/PAMP (microbial)	XAMP (synthetic (agonist))		
TLR1	TLR2	TLR1	*TLR6-TLR1-TLR10*	Nonviral	16	4p14	Cell membrane	Heterodimer of TLR1-TLR2	?	Triacyl lipoproteins	Pam_3_CSK_4_	BCAP, MAL (TIRAP), MyD88, SCIMP	([23], [71], [123], [117], [88], [42], [50], [6], [8])

TLR2	CD14, CD36, dectin-1, GD1a, integrin *β*3, RP105, MBL, LBP, vitronectin, TLR1, TLR2, TLR6 TLR10	TLR1	*TLR2*	Nonviral	24	4q32	Cell membrane	Heterodimer of TLR1-TLR2, homodimer of TLR2-TLR2, heterodimers of TLR2-TLR6 and TLR2-TLR10	Heat shock proteins (HSPs 60, 70, 90), versican, high-mobility group box 1 (HMGB1)	Atypical lipopolysaccharide (LPS), glycoinositolphospholipids, glycolipids, lipoproteins, lipoteichoic acid, zymosan, mannan, peptidoglycan, sporozoite Lipoarabinomannan, porins	Pam_2_CSK_4_, Pam_3_CSK_4_, Amplivant, Lipoamino acids	BCAP, MAL (TIRAP), MyD88, SCIMP	([23], [71], [123], [25], [88], [86], [124], [139], [130], [42], [50], [6], [7], [121], [8])

TLR3	CD14, Mex3B	TLR3	*TLR3*	Viral	23	4q35	Endolysosomal plasma membrane and endoplasmic reticulum	Homodimer of TLR3-TLR3	mRNA, siRNA, and tRNA	Viral double-stranded (ds)RNA	Poly(I : C), Poly(A : U)	SARM, SCIMP, TRIF (TICAM1)	([23], [71], [123], [88], [42], [50], [6], [7], [8], [141])

TLR4	Myeloid differentiation factor 2 (MD-2) or LY96, CD14, CD36, LBP, RP105	TLR4	*TLR4*	Nonviral	22	9q32-33	The cell membrane, endolysosomal plasma membrane, and endoplasmic reticulum	Homodimer of TLR4/MD-2-TLR4/MD-2 Heterodimer of TLR4-TLR6	Heat shock proteins (HSPs 60, 70, 90), amyloid-*β* peptides, oxidized low-density lipoproteins	LPS, type 1, and P fimbriae	Lipid A and related derivatives, e.g., monophosphoryl lipid A Pyrimido-indole (e.g., UM171), Taxol	BCAP, MAL (TIRAP), MyD88, SARM, SCIMP, TRAM (TICAM2), TRIF (TICAM1)	([23], [187], [71], [123], [88], [188], [139], [130], [42], [50], [6], [7], [148])

TLR5		TLR5	*TLR5*	Nonviral	23	1q33.3	Cell membrane	Homodimer of TLR5-TLR5	High-mobility group box 1 (HMGB1)	Flagellin	Recombinant flagellin	MyD88, TRIF (TICAM1)	([23], [71], [123], [88], [50], [6], [8])

TLR6	TLR2, CD36, LBP	TLR1	*TLR6-TLR1-TLR10*	Nonviral	22	4p14	Cell membrane	Heterodimers of TLR2-TLR6 and TLR4-TLR6	Amyloid-*β* peptides, oxidized low-density lipoproteins, versican	Diacyl lipoproteins, lipoteichoic acid, zymosan	M*Φ*-activating lipopeptide 2, synthetic diacylated lipoproteins, Pam2CSK4	BCAP, MAL (TIRAP), MyD88, SCIMP	([23], [71], [123], [117], [88], [130], [42], [50], [6], [8])

TLR7	CD14	TLR7	*TLR7*	Viral	22	Xp22.3	Cell membrane, endolysosomal plasma membrane, and endoplasmic reticulum	Homodimer of TLR7-TLR7	Immune complexes, self RNA	Bacterial and viral (GU-rich) single-stranded (ss) RNA	Imidazoquinoline (such as resiquimod (R848) and imiquimod), thiazoquinoline, oligodeoxynucleotides (ODNs), SM360320, 2-alkoxy-8-hydroxyadenyl derivative, loxoribine, UC-1V150, 3M-012, guanosine analogs	MyD88	([23], [71], [123], [88]; [139], [99], [50], [6], [8], [189])

TLR8		TLR7	*TLR8*	Viral	23	Xp22	Endolysosomal plasma membrane and endoplasmic reticulum	Homodimer of TLR8-TLR8	Immune complexes, self RNA	Bacterial and viral (GU-rich) single-stranded (ss) RNA	Imidazoquinoline (such as resiquimod (R848) and imiquimod), thiazoquinoline, loxoribine, UC-1V150, 3M-012	MyD88	([23], [71], [123], [88], [139], [50], [6], [189])

TLR9	CD14	TLR7	*TLR9*	Viral	22	3p21.3	Endolysosomal plasma membrane and endoplasmic reticulum	Homodimer of TLR9-TLR9	Chromatin IgG immune complexes, self DNA, hemozoin	Viral and bacterial unmethylated cytosine phosphate guanine-dideoxy nucleotide (CpG) DNA, DNA : RNA hybrids	A, B, and C classes of CpG-oligodeoxynucleotides (ODNs)	MAL (TIRAP), MyD88, SCIMP	([23], [71], [123], [88], [99], [50]; [6], [7], [189])

TLR10		TLR1	*TLR6-TLR1-TLR10* (pseudogene in mice)	Nonviral	17	4p14	Cell membrane, endolysosomal plasma membrane, and endoplasmic reticulum	Heterodimers of TLR1-TLR10 and TLR2-TLR10, homodimer of TLR10-TLR10	?	Diacyl lipoprotein, triacyl lipoprotein, viral glycoproteins, double-stranded (ds) RNA	?	MyD88	([23], [71], [123], [117], [88], [86], [124], [50], [6], [8], [189])
